# How Can a Punch Knock You Out?

**DOI:** 10.3389/fneur.2020.570566

**Published:** 2020-10-26

**Authors:** Anders Hånell, Elham Rostami

**Affiliations:** ^1^Section of Neurosurgery, Department of Neuroscience, Uppsala University, Uppsala, Sweden; ^2^Department of Neuroscience, Karolinska Institutet (KI), Stockholm, Sweden

**Keywords:** traumatic brain injury, concussion, boxing, mechanoporation, mechanosensitive (MS) ion channel

## Abstract

Several hypotheses have been put forth over time to explain how consciousness can be so rapidly lost, and then spontaneously regained, following mechanical head trauma. The knockout punch in boxing is a relatively homogenous form of traumatic brain injury and can thus be used to test the predictions of these hypotheses. While none of the hypotheses put forth can be considered fully verified, pore formation following stretching of the axonal cell membrane, mechanoporation, is a strong contender. We here argue that the theoretical foundation of mechanoporation can be strengthened by a comparison with the experimental method electroporation.

## A History of Knockouts

The proportions of the human hand differ from other primates, which tend to have longer fingers. The most likely cause for this is evolutionary pressure aimed at improved manual dexterity to allow better tool use. However, the shape of the human hand also allows it to form a fist which can be used to deliver forceful punches. This was likely very common in prehistoric times, and it has been suggested that the ability to forcefully dominate others also contributed to the evolution of the human hand (1).

Even though the earliest use of punching was likely violent conflict solving, there are mentions of organized competitions in early historical records, such as the Iliad by Homer. Here, there is a description of how Thereon organizes a fight where Epeus uses his brawny fists to strike Euryalus to the ground and wins the first prize of a strong 6-year-old mule. This type of ancient boxing, known as pygmachia, was also practiced in the Olympic games of the ancient era. The tradition continued in the Roman Empire and then gradually changed over the centuries to finally evolve into modern-day boxing.

While the tradition of fighting solely with punches appears to have been confined in the Middle East and Europe, there are numerous examples of martial arts that combine punches with kicks and throws from around the world. These practices have now combined into a large-scale entertainment industry, even though known symptoms of head punches include permanent brain damage and death.

## Symptoms of a Punch

A strike to the head which does not lead to unconsciousness may still induce a state characterized by reduced reaction speed and confusion. This is colloquially referred to as being groggy, since it resembles a person that has had too much grog, an alcoholic beverage. The gait may also be affected, and the afflicted person is then said to have developed spaghetti legs. This is dangerous for a boxer, as it reduces the ability to defend against further punches.

A harder strike can cause a loss of consciousness, typically with an almost instantaneous onset. By observing video sequences of knockouts, it can be concluded that the target of the punch is, in most cases, unconscious before a follow-up punch can land. This usually leads to a complete loss of muscle tone, and the boxer falls to the floor. Fortunately, consciousness is typically regained spontaneously within a few minutes or less. A loss of consciousness from which the person rapidly recovered was termed commotio cerebri (a shaken brain) by early researchers. They distinguished this from contusio cerebri (a bruised brain), where function was lost and never regained (2, 3). This is also seen in boxing, where knocked out boxers sometimes never regain consciousness and succumb to their injuries.

But regardless of whether consciousness is regained or not, it is clear that brain regions responsible for maintaining consciousness must be impaired by the force of the incoming blow.

## The Biomechanics of a Knockout

**Figure 1:**
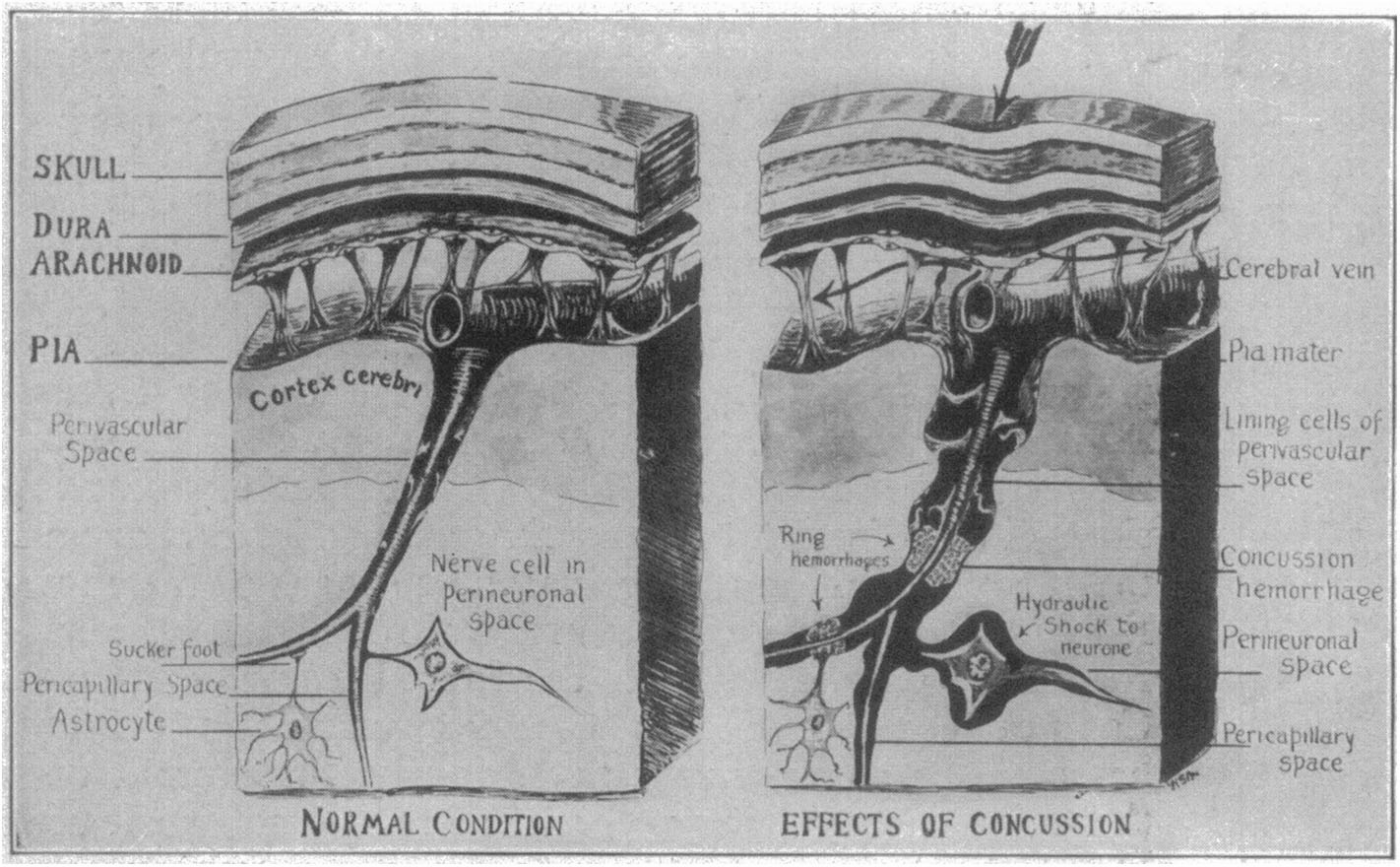
Schematic of Cassasa's hypothesis of concussion described by Martland in 1928, which he attributed to Charles B. Cassasa. The first step is an indentation of the skull caused by a mechanical force. A hydrostatic pressure wave then travels from the subarachnoid space and along the perivascular space to produce ring hemorrhages and hydrostatic shock to the neurons. Reproduced with permission from Martland (4).

For a punch to damage the brain, the force from the impact must somehow be transferred through the skull and into the brain tissue. An early theory was proposed by Charles B Cassasa and reported by Martland in 1928 (4). This theory suggests that the impact leads to a depression of the skull that causes a hydrostatic pressure pulse in the subarachnoid space, which then is transferred through the perivascular space to reach deeper regions of the brain (Figure 1). The assumption that deformation of the skull is a key component of a concussion was, however, contradicted by the work of Denny-Brown and Russel in the 1940s, that demonstrated the importance of head movement (5). Acceleration of the head leads to stretching of the brain tissue itself, which makes it important to consider the direction of the incoming blow and the way it makes the head move.

Retrospective analysis of boxing knockouts has revealed that they typically are caused by a hook to the side of the jaw which causes a rotation of the head in the horizontal plane. Uppercuts to the chin may also cause unconsciousness, while straight punches to the face are unlikely to do so (6). This explains the term “glass jaw,” which for over a 100 years have referred to a boxer that is easily knocked out by a punch to the jaw. The weakness is, however, most likely not the jaw itself, but rather results from an inability of the neck muscles to reduce head movement or a failure of the boxer to notice the incoming strike.

It has been suggested that the loss of consciousness is caused by disruption of axons within the ascending reticular activating system (7–9). Work by Ommaya and Gennarelli in 1960s and 1970s (10), on the other hand, suggested that the cerebral cortex is the brain region most sensitive to mechanical trauma. Recent biomechanical modeling also indicates that the tissue strains caused by knockout blows are considerably higher in the cerebral cortex compared with more caudal regions (6). This is consistent with the larger shift in position experienced by the forebrain compared with the brain stem when the head rotates in the horizontal plane. Therefore, even though modern descriptions typically emphasize the brainstem region, it may be premature to rule out, for example, impairment of the claustrum or disruption of thalamo-cortical oscillations, as the cause for boxing knockouts. But regardless of the anatomical location of the impairment, there must be a way for the mechanical forces of the impact to be translated into neuronal dysfunction.

## The Convulsive Hypothesis

According to this hypothesis, the mechanical forces of the impact trigger a depolarization of the neuronal cell membrane, which leads to uncontrolled release of action potentials. Loss of consciousness would then ensue by a mechanism similar to a grand mal epileptic seizure. One of the earliest formulations of this hypothesis was made in 1944 by Walker et al. (11, 12), who appear to have been inspired by the observation of convulsive movements in laboratory animals following experimental traumatic brain injury. In the 1940s, convulsive movements following head injuries in humans were, however, considered to be very rare. This was explained by the failure of the untrained bystanders to notice these transient events, an explanation that was reasonable at the time. The use of video cameras at sport events has changed this though, and contemporary neurologist has ample opportunities to review concussion inducing events in great detail. This has clearly established that convulsive movements do not only occur in rugby and similar sports following knockouts (13–15) but also that this phenomenon appears to be rare in boxing.

The convulsive hypothesis thus has some merits, although it fails to explain the observed symptoms in most cases.

## Vascular Hypotheses

When a knockout is fatal, a CT scan typically reveals extensive cerebral hemorrhage, which makes it reasonable to assume that tearing of blood vessels is the main form of primary injury in this case. Martland hypothesized that a hydrostatic pressure pulse traveling along the perivascular space would tear apart collateral blood vessels, leading to what he termed ring hemorrhages (4). It is, however, now known that brain trauma that leads to brief unconsciousness typically does not cause hemorrhage that is visible on CT scans. Even if it did, it would be irreversible and does not explain the spontaneous return of consciousness.

A temporary reduction in blood flow would, on the other hand, explain the transient nature of the loss of consciousness seen in knockouts. This is the core of the hypothesis named acute compressive anemia, which was described by Trotter in a speech given to the Medical Society of London in 1924 (16). The basic idea is that the impact would depress the cranium leading to a reduction of the intracranial volume which would then limit cerebral blood flow. This theory is, however, not consistent with the requirement for head acceleration and is now widely considered to be incorrect.

There are, however, other potential causes for a reduction in cerebral blood flow, for example, those that have been described for different forms of syncope. This line of reasoning does not include a description of the primary injury though, and it is not clear how the mechanical forces would translate into a reduced blood flow. Furthermore, even though the brain requires a continuous supply of oxygen and nutrients, it does have limited energy reserves that last for a few seconds. Any hypothesis for boxing knockouts that revolve around a reduction in cerebral blood flow would thus have to explain how the loss of consciousness can manifest so quickly.

The introduction of the notion that concussion requires acceleration of the brain tissue reduced the interest in vascular theories, and the focus shifted toward the stretching of axons as a key event.

## Microtubule Breakage

Normal body movements lead to stretching of axons in the peripheral nervous system which can readily adjust their length without damage in this case (17). The rapid stretching that occurs in traumatic brain injury (TBI), on the other hand, has been hypothesized to make axons brittle and cause the breakage of microtubule (18, 19). This would lead to impaired axonal transport, followed by axonal swelling due to accumulation of transport vesicles, and the eventual disconnection of the axon. So far, this has only been demonstrated using *in vitro* models, but if it holds true, it would represent a type of primary injury in concussion. While this mechanism would be important for long-term effects, it is, however, unlikely to explain the acute loss of consciousness since microtubules are not required for the propagation of action potentials. The acute symptoms may therefore be better explained by an impairment of the axonal cell membrane itself or its embedded ion channels.

## Mechanosensitive Ion Channels

Mechanosensitive ion channels are found in several parts of the body, for example, the heart, urine bladder, and inner ear, where they are required for normal physiology. The brain, on the other hand, is not known to rely on mechanosensitive ion channels for normal function. But it is, however, possible that ion channels that normally open in response to changes in membrane potential or ligand binding, could be affected by mechanical strain in extreme circumstances. Neuronal hyperpolarization following opening of such mechanosensitive K^+^ ion channels has been suggested as the cause for loss of consciousness in boxing knockouts (20). For TBI in general, it has been suggested that a role may be played by several other mechanosensitive ion channels, including Piezo receptors, the NMDA receptor, TRP receptors, as well as voltage-sensitive sodium channels (21–25). Mechanosensitive ion channels have also been suggested to play a role in the later stages of TBI, where they may play a role in the development of secondary injury (26, 27).

The mechanical strain caused by the acceleration of the brain is only present for a very brief period of time though, and it would be expected that these ion channels would return to normal almost immediately. However, if a large fraction of the brain's neurons were simultaneously hyperpolarized or depolarized, it can be speculated that it would take a while for the normal firing patterns to reemerge. If this is the case is not known though, and much experimental work remains before the role of mechanosensitive ion channels can be considered exhaustively described and fully verified.

One potential route is to explore the consequences of knocking out or altering genes for mechanosensitive ion channels in animal models. A clear limitation here is that animals are anesthetized before the induction of brain trauma and the removal of highly expressed ion channels may limit preinjury viability. Another approach is to obtain a detailed description for each mechanosensitive ion channel of the mechanical strains required for opening and the ion selectivity as well as the anatomical and subcellular distribution. This could then serve as a basis for computer modeling of how membrane stretch and channel opening affect neuronal networks.

A key point for strengthening this hypothesis would thus be to explain how a brief membrane stretch can impair consciousness on a timescale of seconds to minutes. This would, however, not be a concern for a mechanism that increases cell membrane permeability with a delayed reversibility.

## The Mechanoporation Hypothesis

Pore formation in a cell membrane induced by mechanical forces is referred to as mechanoporation, which primarily has been investigated by using molecular markers to evaluate cell membrane permeability following experimental brain trauma (28, 29). Briefly, the procedure starts by injecting a marker which floods the cerebral extracellular space. Brain trauma is then induced, and the marker diffuses into cells with increased membrane permeability. After a wash-out period, the marker is cleared from the extracellular space but is still present in compromised cells, either because of insufficient time for diffusion out of the cell or because membrane integrity is restored.

If the marker is larger than the pore, it will not be able to pass through it and the radius of the molecular marker can thus be used to estimate a lower bound on the pore size. Several markers have been used, including horseradish peroxidase (HRP) with a radius of 3 nm (28, 30), 3,000 Da dextran-tetramethylrhodamine (TRITC-dextran) with a radius of approximately 1 nm (31, 32), and Lucifer Yellow with a radius of 0.7 nm (31, 33). At least a subset of the pores would thus have to have a radius of at least 3 nm, although some may be larger, and if the pore size distribution is wide, the majority of pores may be smaller.

While influx of molecular marker can be used to estimate the size of the pores, it does, however, not provide any information about the shape of the pores or the orientation of the phospholipids at the edge of the pores. Since it relies on the diffusion of labeled molecules, it can also only give a rough estimate of the kinetics of pore formation and resealing. Recent computer modeling has, however, demonstrate that pores can form in a fraction of a second following cell membrane stretching, although the kinetics of pore closure has not been evaluated (34, 35). Computer modeling also indicates that pore formation within the cerebral cortex inhibits action potential generation (36), consistent with the loss of consciousness without seizures that is typically observed in boxing knockouts. Whether the seizure-like activity that sometimes is observed following knockout can be explained by mechanoporation is, on the other hand, still an open question.

However, passageways through the cell membrane are normally stabilized by proteins, such as aquaporins or ion channels, to avoid exposing the hydrophobic phospholipid tails to water molecules. This makes it important to consider how pores without a protein component are stabilized and for how long they can remain open. While existing mechanoporation studies do not address these issues, there is an independent line of research that does, namely electroporation (36, 37).

**Figure 2:**
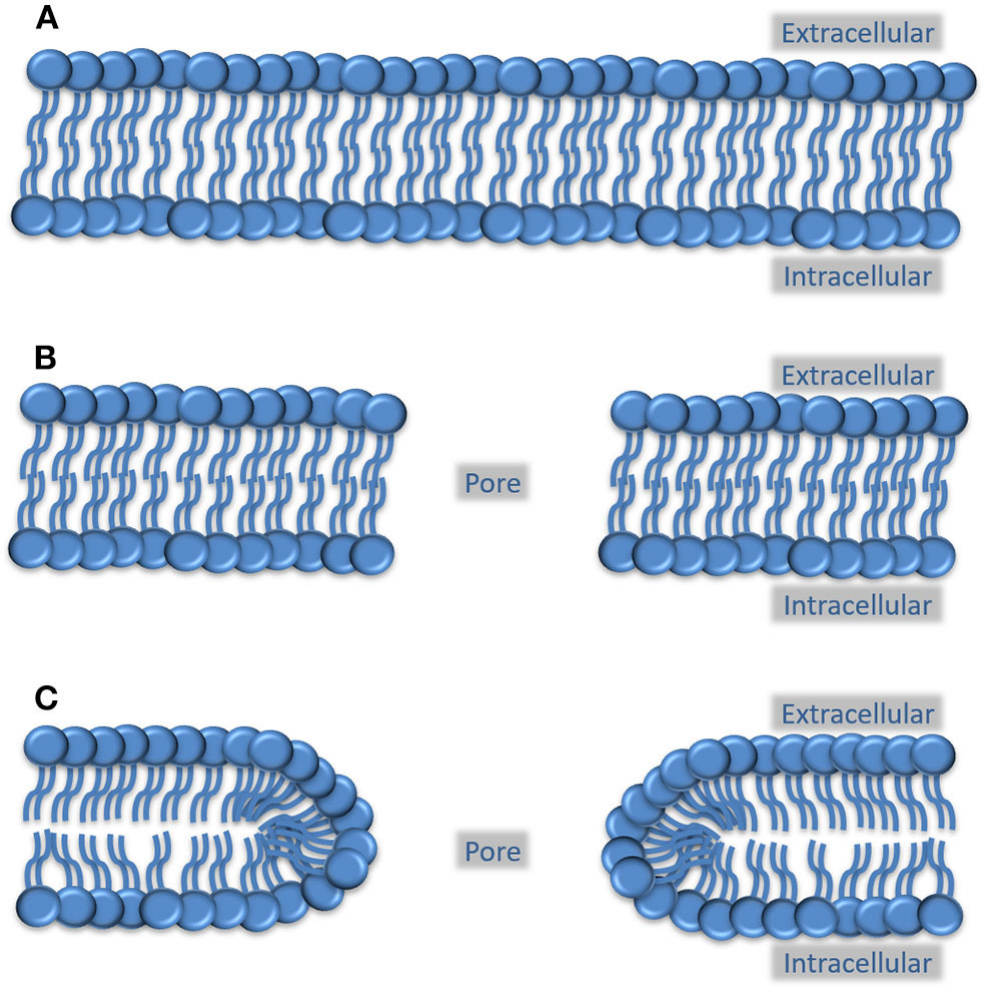
Possible arrangement of phospholipids in a pore created by mechanoporation. **(A)** Preinjury cell membrane, where the circles indicate the hydrophilic phospholipid head groups and the lines indicate the hydrophobic phospholipid tails. **(B)** Phospholipid configuration in a hydrophobic membrane pore, where the hydrophobic tails face the edge of the pore. This type of pore is thought to be highly unstable and close in a fraction of a second. **(C)** Phospholipid configuration in a hydrophilic pore. Here, the polar head groups face the edge of the pore which prevents the pore from immediately collapsing. The crowding of the fatty acid tails at the edges does, however, causes instability and will, within minutes, lead to the closure of the pore.

In the experimental electroporation method, an electrical current is passed through a cell suspension, which induces cell membrane pores that stay open long enough to allow genetic material to diffuse into the cells. According to current theories in this field, the pores can either be hydrophobic or hydrophilic (38), depending on the orientation of the phospholipids within the membrane (Figure 2). Based on what is known from kinetic studies, hydrophobic pores are inherently unstable and closes in a fraction of a second while hydrophilic pores does so in a timeframe of seconds to minutes (39, 40).

It can be argued that once a pore has been formed, the kinetics of resealing should be independent of the nature of the forces that lead to its creation. If this is true then porated neurons in a knocked-out boxer would be resealed within minutes, leading to the return of consciousness. Thus, the timeframe for both the formation and the spontaneous closure of the pores are a good match for the observed symptoms. Mechanoporation could potentially also be the initial trigger for long-term consequences of a knockout, for example, if Ca^2+^ influx activates proteases or if an efflux of intracellular proteins triggers a neuroinflammatory response.

The experimental evidence for mechanoporation is, however, so far indirect and relies on influx of marker substances in animal models and *in silico* modeling. More direct evidence, such as visualization of actual pores, is still not available and more work remains before mechanoporation can be considered fully verified.

## Benefits of Understanding Knockouts

It may be that seeing a person being knocked out, and then rapidly regaining consciousness, is so common that it is taken for granted. But from the above description, it should be clear that this sequence of events is difficult to explain on a cellular and molecular level. Compared with concussion in general, the patient characteristic and mechanical forces in the boxing knockout are very homogeneous and may therefore serve as a suitable starting point. Admittedly, the timescale of the primary injury precludes any medical intervention and future therapeutics would therefore have to target secondary injury processes. A fully verified and detailed description of the primary injury in boxing knockouts, and concussion in general, would be very useful though. It could potentially make it possible to define a safe level of mechanical strain on brain tissue, which would aid in the design of protective equipment and the regulation of sports. An increased understanding of the pathophysiological mechanism would also increase the chances of finding better treatments for patients with postconcussive syndrome, chronic traumatic encephalopathy, and diffuse axonal injury. And perhaps a better understanding of how consciousness is lost may be a first step toward understanding consciousness itself.

## Author Contributions

AH drafted the original manuscript. ER contributed novel ideas, insights, and knowledge to the final manuscript. Both authors contributed to the article and approved the submitted version.

## Conflict of Interest

The authors declare that the research was conducted in the absence of any commercial or financial relationships that could be construed as a potential conflict of interest.
